# Recognizing and Addressing the Contraceptive Hesitancy-Acceptability Continuum: Adopting Lessons Learned From the Immunization Field

**DOI:** 10.9745/GHSP-D-24-00220

**Published:** 2024-12-20

**Authors:** Madeleine Short Fabic, Amy Ong Tsui

**Affiliations:** a U.S. Agency for International Development, Washington, DC, USA.; b Johns Hopkins Bloomberg School of Public Health, Baltimore, MD, USA.

## Abstract

We propose a new framework that builds from vaccine hesitancy concepts and findings for the family planning community to better conceptualize, measure, and address the major drivers of contraceptive hesitancy and acceptability.

## Key Messages

- Given the similarities the family planning (FP) and immunization fields share, we recommend that the FP community consider adopting vaccine hesitancy lessons to take a promising new measurement, investigation, and programming direction.
- Specifically, building from the 5C scale for measuring vaccine hesitancy—with its components of confidence, constraints, complacency, calculation, and collective responsibility—the FP community can develop and use a new framework to identify and track the major drivers of contraceptive-related behaviors at individual- and community-levels, dubbed the “contraceptive hesitancy-acceptability continuum.”
- The continuum helps to reorient FP’s measurement approach from a largely binary focus on contraceptive use/non-use, need/met need to a multifaceted focus on the psychosocial context in which individuals make and act on decisions about their reproductive lives. Ultimately, this measurement reorientation will better position FP programs to help address individuals’ sexual and reproductive health needs, aspirations, and rights.

See related article by McDougal et al.

## INTRODUCTION

Family planning (FP) programs are generally designed to support individuals and couples in enacting their pregnancy prevention intentions and are often premised on an ideal that everyone who wants to avoid pregnancy can be protected by voluntary, safe, and effective contraception, which includes fertility awareness-based methods (i.e., standard days, 2-day, and lactational amenorrhea methods); barrier methods (i.e., male and female condoms, diaphragms and spermicides, and cervical caps); hormonal methods (i.e., pills, injectables, implants, combined patch, vaginal ring, and hormonal intrauterine device [IUD]); and other nonhormonal methods (i.e., copper IUD, vasectomy, and tubal ligation).^1^ As program managers and policymakers direct limited resources toward achieving that ideal, data are needed to inform the investments, including data to identify numbers and types of contraceptive users and potential users, as well as barriers to use. Two key indicators—contraceptive prevalence and unmet need—provide some insight into the numbers and types of contraceptive users and potential users; however, both have limitations. For example, contraceptive prevalence tells nothing about method satisfaction or barriers to use. And the proxy indicator for potential users, “unmet need for family planning,” is not predictive of individual-level demand for contraception or use.^2^ Furthermore, FP indicators operate on a dichotomy (contraceptive user, yes/no; unmet need, yes/no), which limits deeper understanding of contraceptive use and barriers to use, let alone barriers to method switching or discontinuation.

To overcome these long-standing data limitations, the FP field can adopt lessons learned from the immunization field’s efforts to measure and understand vaccine hesitancy, which build from established theoretical frameworks, including the Health Belief Model and Theory of Planned Behavior.^3^^–^^5^ Specifically, by developing and using a new framework, the “contraceptive hesitancy-acceptability continuum,” the FP community can identify the major drivers of contraceptive-related behavior at individual and community levels. We propose framing this new concept as the contraceptive hesitancy-acceptability continuum rather than just “contraceptive hesitancy” to minimize inferences about what an individual’s contraceptive behavior “should” be. “Hesitancy” as a public health concept is more likely to be understood as a “problem to be overcome” compared to “acceptability,” which is perceived as more neutral.

We hypothesize that this framework can better inform programs such that salient barriers to contraceptive access and use can be reduced, each individual’s reproductive goals can be achieved, and the burden of unintended pregnancy can be minimized.

We hypothesize that the contraceptive hesitancy-acceptability continuum framework can better inform programs such that salient barriers to contraceptive access and use can be reduced.

Before further describing our hypothesis, let us first address an important distinction—that contraception and vaccination coverage are not equivalent. Pregnancy is not a disease. Unlike vaccination, contraception is not always a universal good, although its widespread normative use offers many social and individual health benefits. Our interest in applying a vaccine hesitancy lens toward our understanding of contraceptive behaviors is to build from immunization’s lessons to better appreciate individual’s psychosocial and behavioral reactions and responses to efficacious contraceptive technologies—inclusive of those methods that require hormones, a device, or a procedure as well as those that do not (i.e., fertility awareness-based methods that are based on reproductive biology).^6^

We provide the rationale for developing and using a new framework to measure and understand the contraceptive hesitancy-acceptability continuum, which builds from vaccine hesitancy concepts and findings. An accompanying article in *GHSP* shared results and perspectives from a recent literature review led by the Agency for All project consortium, which confirmed the applicability of hesitancy concepts to contraceptive-related attitudes and behaviors in sub-Saharan African geographies.^7^ Together, these 2 articles have identified a promising direction for FP measurement, investigation, and programming.

## WORLD HEALTH ORGANIZATION STRATEGIC ADVISORY GROUP OF EXPERTS ON IMMUNIZATION RECOMMENDATIONS

In the mid-2010s, the immunization field moved from the binary categories of “vaccinated/unvaccinated” or “pro-vaccine/anti-vaccine” to recognize a new concept: vaccine hesitancy. As defined by the World Health Organization’s (WHO) 2014 Strategic Advisory Group of Experts on Immunization (SAGE)^8^:

*Vaccine hesitancy refers to delay in acceptance or refusal of vaccines despite availability of vaccine services. Vaccine hesitancy is complex and context specific, varying across time, place and vaccines. It is influenced by factors such as complacency, convenience and confidence. … Vaccine hesitancy occurs on the continuum between high vaccine demand and complete vaccine refusal… At both the individual and community level, if vaccine hesitancy is present, it undermines personal and community responsibility for immunization.*

SAGE also recognized the linkages with FP noting^8^:

*Given that reproductive health decisions are a behavioral phenomenon like vaccine decisions… [approaches] to address hesitancy surrounding the acceptance of reproductive health interventions (segmentation of the population to find the reproductive health hesitant subgroups, diagnosis of the major causes of hesitancy in these subgroups and then tailoring the intervention to address the causes) might lead to further improvements in uptake, as has been seen with vaccine hesitancy.*

Ten years have passed since SAGE identified these commonalities; however, the FP field has not yet adopted and adapted vaccine hesitancy lessons.

## VACCINATION AND CONTRACEPTION SIMILARITIES

Immunization lessons are applicable to FP due to the 2 fields’ many similarities, including the following 3 technology characteristics.

1. **They are highly efficacious.** Vaccines prevent disease. Contraceptives prevent unwanted pregnancy. Both prevention technologies are highly efficacious based on decades of biomedical and clinical research.
2. **They have minimal side effects.** As with today’s vaccines, today’s contraceptives have minimal side effects that are generally well tolerated. Side effects for both tend to be temporary and short-lived.
3. **Their uptake depends on behavioral phenomena.** Vaccine and contraceptive uptake both rely on behavioral phenomena—individuals’ and groups’ knowledge, attitudes, beliefs, motivations, perceptions, norms, relationships, cognitions, and emotions. Not all individuals at risk of disease accept vaccination, just as not all individuals at risk of unintended pregnancy accept contraception.

Finally, there is an additional hypothesized way in which immunization and contraception are similar—they can confer herd immunity if widely adopted. Herd immunity is the concept that if enough people in a population develop immunity—typically through immunization, sometimes by widespread infection—then the vulnerable members of that population will be protected, too.^9^ This infectious disease concept may be applicable to contraception. Behavioral epidemiologists posit that social epidemics can arise through the diffusion of beneficial elements, with the speed of the spread related to the connectivity among individuals.^10^ It has been hypothesized that contraception confers immunity from unintended pregnancy, and social diffusion of contraception as a behavioral norm confers herd immunity to the population.^11^ That is, as contraception becomes more widely known, accessible, acceptable, and used in a society, the rate of unintended pregnancy declines and the risk and consequences of unintended pregnancy diminish for everyone in that society, including those who remain vulnerable. Through these mechanisms, contraception acts as a “social vaccine.”^12^ Aggregate protection of the “herd” includes diminished risk exposures to poor maternal and child health, household poverty, and gender inequality.^13^

There are, unquestionably, limits to the parallels. Contraceptive use must unequivocally be a choice; reproductive autonomy is a human right. Vaccination, in contrast, may ethically be mandated for the good of the community. Additionally, efforts to recognize likenesses between contraception and vaccination must avoid feeding misinformation and disinformation linking immunization with coercive fertility control. Furthermore, regardless of the negative health impacts of unintended pregnancy,^14^^–^^16^ pregnancy is not a disease; any correlation between vaccination and contraception must be tempered with this recognition.

## CONTRACEPTIVE HESITANCY-ACCEPTABILITY CONTINUUM FRAMEWORK

**FIGURE 1:**
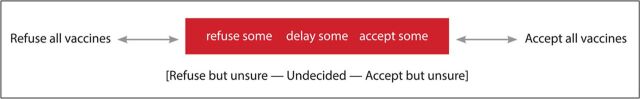
Spectrum of Vaccine Hesitancy Source: Violette and Pullaguara.^17^

Vaccine hesitancy programming and research have provided a spectrum of vaccination outcomes (Figure 1); so too should contraceptive programming and research.^17^ The spectrum is wide and heterogenous, spanning individuals who will refuse all contraceptives no matter what to those who will adopt any method to protect against an unwanted pregnancy. Between these 2 endpoints are many types of users and non-users (both never users and former users), including individuals who are hesitant to begin using a contraceptive method after the birth of a child, a miscarriage or abortion, or another life event (“refuse but unsure”); are unsure whether potential contraceptive side effects outweigh the risks of unintended pregnancy (“undecided”); and use contraception but are dissatisfied with their method and/or hesitant to switch to another (“accept but unsure”). Where an individual sits on the spectrum will likely change over time and with different life experiences; hesitancy-acceptability to initiate may look very different than hesitancy-acceptability to continue.

Vaccine hesitancy may vary by specific vaccine (e.g., polio, human papillomavirus, and COVID-19); administration route (e.g., oral, intranasal, and injection); and vaccine type (e.g., attenuated, inactivated, and mRNA). Similarly, contraceptive hesitancy-acceptability may vary by specific methods (e.g., injectables, pills, and condoms); administration route (e.g., oral, injection, vaginal, and surgical); method type (e.g., hormonal, fertility awareness-based, and short- or long-acting); and user control (e.g., provider-controlled, female-controlled, and male-controlled). A substantial benefit of the continuum is that it allows for a deeper understanding of contraceptive hesitancy-acceptance in nonbinary terms, representing a major departure from existing FP indicators. Moreover, the continuum helps to reorient FP’s measurement approach from a largely binary focus on contraceptive use/non-use, need/met need to a multifaceted focus on the psychosocial context in which individuals make and act on decisions about their reproductive lives. Communal acceptability of contraceptives arises to serve as a positive social norm that supports an individual’s reproductive agency, whether that be to use contraception or not.

A substantial benefit of the continuum is that it allows for a deeper understanding of contraceptive hesitancy-acceptance in nonbinary terms.

To develop the necessary contraceptive hesitancy-acceptability measures, the FP field can embrace questions that include psychosocial scales. To begin, the field can transform the contraceptive intention question long used in population-based household surveys, “Do you think you will use a contraceptive method to delay or avoid pregnancy at any time in the future? [yes/no/unsure]”, into a scale. For example, intention responses can be expanded to no, probably no, undecided, probably yes, and yes. Such a change can help elucidate the spectrum of contraceptive intent.

Second, the field can build from the vaccination community’s 5C scale—confidence, constraints, complacency, calculation, and collective responsibility—for measuring vaccine hesitancy. While there are myriad hesitancy scales, the 5C scale is widely used and cited and includes all aspects of vaccine hesitancy available in the literature.^5^ Based on social and individual dimensions of vaccine hesitancy, the scale provides a validated short set of questions to monitor hesitancy and includes a sample study protocol for adaptation and translation.^18^ To demonstrate the 5C scale’s applicability to FP, we have provided illustrative questions for adaptation and validation (Table).^5^

Importantly, the 5C scale includes perceptions of service-related factors, such as convenience and comfort with health providers, in recognition that the hesitancy-acceptability continuum is influenced by demand- and supply-side factors.^19^ Additionally, unlike many existing FP measures that are assessed only among women of reproductive age, the 5C scale has applicability to people of all gender identities. This widespread applicability is important given the pervasive influence of social norms, including gender norms, on contraceptive attitudes and behaviors.^20^ By measuring the hesitancy-acceptability continuum among the broader population, including and beyond women of reproductive age, the 5C scale can provide individual-, community-, and population-level insights and estimates of underlying attitudes influencing contraceptive behaviors.

The 5C scale can provide individual-, community-, and population-level insights and estimates of underlying attitudes influencing contraceptive behaviors.

**FIGURE 2:**
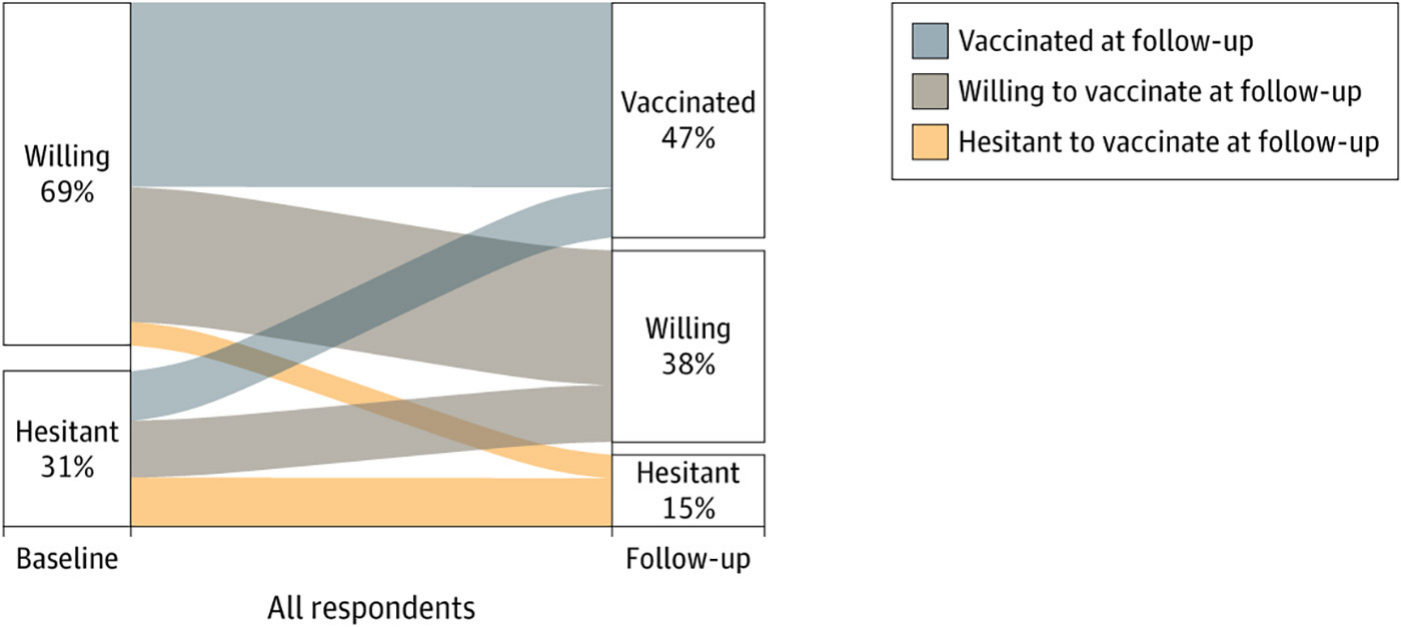
Vaccination Hesitancy Over Time^a^ in the United States ^a^Among 3,349 U.S. survey respondents at baseline (August 9 to December 8, 2020) and follow-up (March 2 to April 21, 2021). Source: Seigler et al.^22^

To date, the FP field’s largest and longest-enduring population-based household survey program, the Demographic and Health Surveys, has avoided the use of scales. Despite this limitation, other surveys, including Performance Monitoring for Action (PMA), afford opportunities to test new questions and take more novel approaches to address FP data needs. For example, the longitudinal data offered by PMA allows for monitoring of the fluidity of contraceptive intentions and uptake.^21^ This type of monitoring is akin to recent analyses of COVID-19 vaccination intention fluidity undertaken by other researchers (Figure 2).^22^ PMA could incorporate information on the 5Cs into their longitudinal cohort data collection activities, which would allow for measurement of individual- and population-level contraceptive hesitancy-acceptability trends and their connections with contraceptive behaviors. Similarly, social behavior change programs could adopt and adapt the 5Cs as a systematic, short form set of questions to inform programmatic direction, monitoring, and evaluation. Using such information, FP programs could better identify and address psychosocial issues impacting the spectrum of contraceptive initiation, continuation, and repeated use as well as non-use.

The Agency for All project, with funding from the U.S. Agency for International Development, has begun to explore the applicability of the 5C scale to FP. Findings from their recent literature review are encouraging; they show thematic clustering of contraceptive hesitancy and acceptability around the 5Cs, indicating conceptual linkages.^23^ We commend Agency for All’s efforts and await results from their ongoing field research. In the meantime, we recommend that others interested in this new framework read the accompanying commentary in *GHSP,* which synthesizes Agency for All’s review findings.^7^

## CONCLUSION

The FP and immunization fields share many similarities. Contraceptives and vaccines are both highly efficacious technologies, have minimal side effects, and their uptake depends on behavioral phenomena. In the mid-2010s, the immunization field moved from the binary vaccinated/unvaccinated to a new concept: vaccine hesitancy. In the mid-2020s, the FP field should make a comparable shift. Ten years after the SAGE recommendation and 30 years after the International Conference on Population and Development Programme of Action articulated FP’s “focus on the needs, aspirations and rights of individual women and men,”^24^ it is never too late to take meaningful action to adapt and improve. Specifically, building from the 5C scale for measuring vaccine hesitancy, the FP community can better conceptualize, measure, and address the major drivers of contraceptive hesitancy and acceptability. Ultimately, these efforts will better position FP programs to help address individuals’ sexual and reproductive health needs, aspirations, and rights.

**TABLE.** 5C^a^ Scale Vaccine Hesitancy Short-Form Questions and Possible Questions for Contraceptive Hesitancy-Acceptability

	**Short-Form Questions** ^b^	
	**Original Vaccine Hesitancy**	**Adapted Contraceptive Hesitancy-Acceptability**
Confidence	**I am completely confident that vaccines are safe.** Vaccinations are effective. Regarding vaccines, I am confident that public authorities decide in the best interest of the community.	**I am confident that contraceptives are safe.** Contraceptives are effective. Regarding contraceptives, I am confident that health providers prioritize my best interest.
Constraints	**Everyday stress prevents me from getting vaccinated.** For me, it is inconvenient to be vaccinated. Visiting the doctor makes me feel uncomfortable; this keeps me from being vaccinated.	**Circumstances in my life prevent me from using contraceptives.** For me, it is difficult to access contraceptives. Visiting health providers makes me feel uncomfortable; this keeps me from using contraception.
Complacency	**Vaccination is unnecessary because vaccine-preventable diseases are not common anymore.** My immune system is so strong; it also protects me against diseases. Vaccine-preventable diseases are not so severe that I should be vaccinated.	**Contraception is unnecessary because I am unlikely to have an unplanned pregnancy.** My body is unlikely to be fertile; I do not need to worry about unplanned pregnancy. Contraception is unnecessary because an unplanned pregnancy would not be a problem for me.
Calculation	**When I think about getting vaccinated, I weigh benefits and risks to make the best decision possible.** For each and every vaccination, I closely consider whether it is useful for me. It is important for me to fully understand the topic of vaccination before I get vaccinated.	**When I think about using contraceptives, I weigh benefits and risks to make the best decision possible.** For each and every form of contraception, I closely consider whether it will be suitable for me. It is important for me to fully understand the topic of contraception before I use contraception.
Collective responsibility	**When everyone is vaccinated, I don’t have to get vaccinated, too.** I get vaccinated because I can also protect people with a weaker immune system. Vaccination is a collective action to prevent the spread of diseases.	**It is my partner’s responsibility to contracept, so I don’t have to.**^c^ I use contraception so I can take better care of my family. Contraception is an action that can improve the well-being of individuals, families, and communities.

^a^ Instructions for the 5C are: “Please evaluate how much you disagree or agree with the following statements.” (1=strongly disagree, 2=moderately disagree, 3=slightly disagree, 4=neutral, 5=slightly agree, 6=moderately agree, 7=strongly agree). The items used for the short scale are in bold font.

^b^ Items related to each of the short-form questions are bulleted beneath each short-form question. Adapted questions are provided for illustrative purposes only; they must be tested and validated.

^c^ Partner responsibility and collective responsibility do not involve equivalent actors but have in common a perception of shared responsibility for others’ health (e.g., the degree to which an individual perceives vaccination or contraception as part of their responsibility to others).

## Notes

**Peer Reviewed**

**First Published Online:** November 25, 2024.
